# Treatment of Type 2 Diabetes by Free Fatty Acid Receptor Agonists

**DOI:** 10.3389/fendo.2014.00137

**Published:** 2014-08-28

**Authors:** Kenneth R. Watterson, Brian D. Hudson, Trond Ulven, Graeme Milligan

**Affiliations:** ^1^Molecular Pharmacology Group, Institute of Molecular, Cell and Systems Biology, College of Medical, Veterinary and Life Sciences, University of Glasgow, Glasgow, UK; ^2^Department of Physics, Chemistry and Pharmacy, University of Southern Denmark, Odense, Denmark

**Keywords:** diabetes, FFA receptor, insulin, incretin, inflammation

## Abstract

Dietary free fatty acids (FFAs), such as ω-3 fatty acids, regulate metabolic and anti-inflammatory processes, with many of these effects attributed to FFAs interacting with a family of G protein-coupled receptors. Selective synthetic ligands for free fatty acid receptors (FFA1-4) have consequently been developed as potential treatments for type 2 diabetes (T2D). In particular, clinical studies show that Fasiglifam, an agonist of the long-chain FFA receptor, FFA1, improved glycemic control and reduced HbA1c levels in T2D patients, with a reduced risk of hypoglycemia. However, this ligand was removed from clinical trials due to potential liver toxicity and determining if this is a target or a ligand-specific feature is now of major importance. Pre-clinical studies also show that FFA4 agonism increases insulin sensitivity, induces weight loss, and reduces inflammation and the metabolic and anti-inflammatory effects of short chain fatty acids (SCFAs) are linked with FFA2 and FFA3 activation. In this review, we therefore show that FFA receptor agonism is a potential clinical target for T2D treatment and discuss ongoing drug development programs within industry and academia aimed at improving the safety and effectiveness of these potential treatments.

## Introduction

In 2013, 382 million people worldwide were characterized as diabetic patients with around 90% of patients diagnosed with type 2 diabetes (T2D), a metabolic disorder intrinsically linked with obesity (1). T2D is defined by insulin resistance in peripheral tissues, such as the liver and muscle, and a loss of pancreatic beta-cell function, resulting in insufficient insulin secretion (2), and constitutes a risk factor for health issues including cardiovascular disease, impaired wound healing, blindness, and renal failure (1). Although T2D can sometimes be controlled through strict diet regulation, a large number of patients require clinical therapies. Current treatments, such as metformin, sulfonylureas, glucagon-like peptide-1 (GLP-1) receptor agonists, and dipeptidyl peptidase-4 (DPP-4) inhibitors, are deployed primarily to either improve insulin secretion, peripheral insulin sensitivity, or both (3). However, there remains a demand for distinct, safe, and effective treatments for T2D, with the current therapies often associated with side effects including hypoglycemia and weight gain. Naturally occurring free fatty acids (FFAs) found in the diet, including ω-3 fatty acids, have profound effects on metabolic and inflammatory processes associated with T2D, although the molecular basis for these effects are complex and incompletely understood (4). FFAs are classified based upon their chain length, such that short chain fatty acids (SCFAs) have 1–6 carbon atoms; medium chain fatty acids (MCFAs) contains 7–12 carbon atoms; and long-chain fatty acids (LCFAs) contain more than 12 carbon atoms (4). Many of the biological effects of FFAs have now been attributed, at least in part, to FFAs interacting with a group of G protein-coupled receptors (GPCRs) designated the FFA receptors. The most well-characterized FFA receptors are the two LCFA-specific receptors, FFA1 and FFA4, and the SCFA-specific receptors FFA2 and FFA3. FFA receptor agonism, particularly of the FFA1 receptor, has subsequently been shown to have beneficial metabolic effects (4). Consequently, a number of ongoing industrial and academic programs are focused upon developing potent and selective synthetic agonists of FFA1. Although currently less developed, activation of each of FFA2, FFA3, and FFA4 has also been suggested to have potential benefits for metabolic function. In this review, we will therefore discuss the potential of FFA receptor agonists as novel clinical treatments for T2D.

## FFA1

**Figure 1:**
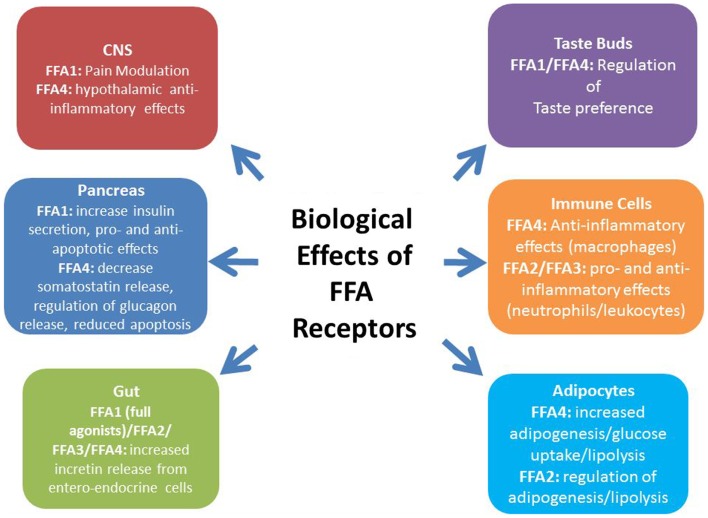
**The biological effects of FFA receptors**. Dietary FFAs, such as ω-3 fatty acids from fish oils and SCFAs derived from the fermentation of dietary fiber, have profound effects on metabolic and inflammatory processes associated with obesity and T2D. These effects have, at least in part, been attributed to the activation of free fatty acid receptors (FFA1–4), leading to a great deal of interest in the development of synthetic FFA receptor agonists for the treatment of metabolic disease. Agonism of the long-chain FFA receptor FFA1, the most fully characterized of these receptors, improves glucose-stimulated insulin secretion from the pancreas. Additionally, full agonists of this receptor increase incretin release from the gut, thereby indirectly increasing pancreatic insulin secretion, as well as improving systemic insulin sensitivity and promoting satiety. Agonism of another long-chain FFA receptor, FFA4, is associated with incretin release from the gut, as well as an anti-inflammatory effect on macrophages that, in turn, may improve systemic insulin sensitivity. In the pancreas, FFA4 is associated with reduced cell apoptosis and FFA4 has recently been detected in alpha and delta cells and regulates glucagon and somatostatin release, respectively. Both FFA1 and FFA4 have been detected in taste buds although the full implications of this in relation to obesity remain to be determined. The SCFA receptors, FFA2/FFA3, have recently been linked with the beneficial metabolic effects associated probiotics within the gut. Both receptors have been linked with incretin release from enteroendocrine cells, as well as both systemic anti- and pro-inflammatory effects. However, due to conflicting results using receptor-specific knockout models and a limited selection of pharmacological tools, more work is required to elucidate the physiological effects of FFA2 and FFA3 agonism.

FFA1, activated by various saturated (e.g., palmitic acid, C16:0), mono-unsaturated (e.g., oleic acid, C18:1), and polyunsaturated long-chain FFAs (e.g., linoleic acid, C18:2) (Table 1), is a G_q_/_11_-coupled GPCR predominantly expressed in pancreatic beta cells that is associated with increased glucose-stimulated insulin secretion (GSIS) (4–6) (Figure 1). FFA1 is also expressed by various enteroendocrine cells where it regulates the release of incretin hormones such as glucagon-like peptide-1 (GLP-1), an insulinotropic, anorectic peptide that reduces gastric emptying and motility, as well as cholecystokinin (CCK), shown to regulate pancreatic secretion, inhibit gastric motility, and reduce energy intake (7–10) (Figure 1). FFA1 is also present within the central nervous system (CNS) (11, 12) although whether neuronal FFA1 contributes to the regulation of glucose homeostasis remains to be fully determined (Figure 1). FFA1 expression has also been reported in glucagon-producing alpha cells within the pancreas, although this remains controversial (13–17). FFA1 expression has also been well characterized in taste buds where it mediates, in part, taste preference for fatty acids, although the significance of this, and possible effects of pharmacological activation or blockade, remains to be fully elucidated (18, 19) (Figure 1).

**Table 1:** **FFA receptor agonists for the treatment of T2D**.

FFA receptor	Agonists	Metabolic effects	Clinical trial status
FFA1	**Natural ligands:** palmitic acid, oleic acid, linoleic acid **Synthetic ligands:** GW9508, TAK-875/Fasiglifam, AMG-837, AM-1638, AM-5262, LY2881835, JTT-851, P11187, TUG-469, TUG-424, TUG-770, AS2575959, DS-1558	Improved fasting hypoglycemia and glucose tolerance in diabetic animal models Increased GSIS Increased incretin release (full agonists: AM-1638, AM-5262, LY2881835) No associated hypoglycemia in normoglycemic rats	**TAK-875/Fasiglifam (Takeda):** phase I/II trials showed reduced blood glucose levels, increased insulin levels, 1.2–1.4% reduction in HbA1c levels with no associated weight gain/hypoglycemia in T2D patients. Removed from phase III trials due to potential liver toxicity **AMG-837 (Amgen) and LY2881835 (Eli Lilly):** removed from phase I trials due to toxicity **JTT-851 (Japan Tobacco):** currently in phase II trials **P11187 (Piramal):** currently in phase I trials
FFA2	**Natural ligands:** acetate (preferred), propionate, butyrate **Synthetic ligands:** AMG7703/4-CMTB, Euroscreen compounds, compounds 1 and 2	Improved glucose uptake Decreased colon motility/contractility Increased GLP-1 secretion Inhibition of leukocyte activation	No agonists currently in clinical trials
FFA3	**Natural ligands:** propionate (preferred), butyrate, acetate	Increased GLP-1 secretion	No agonists currently in clinical trials
	**Synthetic ligands:** Arena Pharmaceuticals series	
FFA4	**Natural ligands:** α-linolenic acid (αLA), docosahexanoic acid (DHA) **Synthetic ligands:** GW9508, NCG21, NCG46, TUG-891	Protection against diet-induced obesity Improved insulin sensitivity and glycemic control Increased GLP-1 release Increased insulin secretion (largely attributed to GLP-1 release) Reduced inflammation	No compounds currently in clinical trials although a number of companies have patented FFA4 agonists for the treatment of T2D (Banyu Pharmaceutical, Metabolex, Kindex Therapeutics, Pharma Frontier)

*Table 1 illustrates the most commonly described natural and synthetic ligands for FFA1-4. The current clinical status of these synthetic agonists for the treatment of T2D is also described*.

## FFA1 and Insulin Secretion

Acute FFA-mediated insulin secretion from isolated human and rodent islets involves amplification of the second phase of GSIS (5, 6, 15, 20). This is reduced by approximately 50% in FFA1-null mice, with the remaining effect attributed to intracellular metabolism of FFAs (5, 6, 15, 20). In contrast, transgenic overexpression of FFA1 under the control of the mouse insulin II promoter prevents development of hyperglycemia and improves insulin secretion and glucose tolerance in diabetic mouse models (21). As anticipated from this, GW9508, a synthetic FFA1 agonist (Table 1) stimulated GSIS in pancreatic MIN6 cells (22). FFA1 gene expression is also reduced under glucolipotoxic conditions in rats and in islets from T2D patients while a rare mutation in the human FFA1 gene is associated with attenuated lipid-mediated enhancement of GSIS (23–25). The effects of FFA1 on pancreatic beta cell viability, however, has been controversial, with pancreatic-specific FFA1 overexpression associated with disrupted islet morphology and impaired beta cell function whereas FFA1 disruption is linked with increased beta cell viability in mice fed on a high-fat diet (HFD) (26). These observations promoted the concept that, at least in the longer term, FFA1 antagonism could be beneficial in the treatment of diabetes. However, most subsequent pre-clinical studies contradict these findings, indicating that FFA1 agonism has no detrimental effects on beta cell viability (16, 20, 21), or even protects beta cells (27–29).

## The FFA1 Agonist Fasiglifam and Insulin Secretion

**Figure 2:**
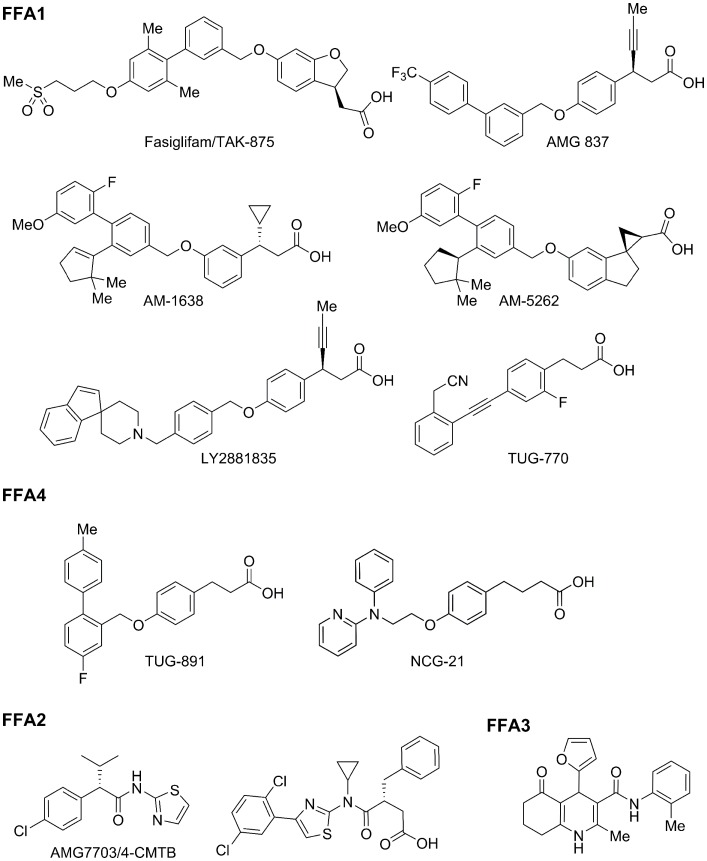
**Representative free fatty acid receptor agonists**. A number of academic and industrial drug programs are aimed at developing FFA receptor agonists for the treatment of T2D. A representative selection of the current range of synthetic agonists that have so far been developed for these receptors are shown.

Although there are currently no FFA1 agonists approved for clinical use, considerable interest developed around Fasiglifam (designated TAK-875 in pre-clinical studies, Figure 2), an orally available FFA1 agonist developed by Takeda (30–32) (Table 1). Completed Phase II clinical trials demonstrated that T2D patients treated with Fasiglifam had reduced blood glucose levels, increased insulin levels, and resulted in a 1.2–1.4% reduction in hemoglobin A1c (HbA1c) levels (32–36) (Table 1). Crucially, although these effects were comparable to current sulfonylurea treatments, Fasiglifam was associated with markedly less side effects, with no significant increases in body weight and a reduced concomitant incidence of hypoglycemia (32–36). This is consistent with pre-clinical data demonstrating that Fasiglifam improved fasting hyperglycemia and glucose tolerance and augmented GSIS in diabetic rat models, with no hypoglycemia observed in normoglycemic rats (31). No changes in insulin resistance have been reported in response to Fasiglifam treatment (37, 38) and Fasiglifam had no effect on glucagon secretion in isolated human islets and did not alter glucagon levels in T2D patients (39). Importantly, prolonged Fasiglifam exposure was also not associated with beta cell dysfunction or apoptosis (31).

## The Effect of Partial vs. Full FFA1 Agonists on Incretin Release from Enteroendocrine Cells

The ability of synthetic FFA1 agonists to induce significant incretin release was recently shown to depend upon whether the compound was a partial or full agonist (8, 10, 40) (Figure 1). In this regard, TAK-875/Fasiglifam had no effect on incretin release from enteroendocrine cells with similar results reported for AMG-837 (Amgen, Table 1; Figure 2) (39). In contrast, Amgen described AM-1638 and AM-5262 (Table 1; Figure 2) as full FFA1 agonists that directly stimulate insulin secretion and promote incretin release from enteroendocrine cells (39, 41, 42) (Figure 1). This incretin-stimulating effect was abolished in FFA1 knockout mice and the effect of AM-1638 on glucose homeostasis was attenuated by the GLP-1R antagonist, Ex(9–39)NH2, indicating a particularly key role for GLP-1 (39). Similarly, LY2881835, a full FFA1 agonist from Eli Lilly (Table 1), increased GSIS, lowered blood glucose levels, and increased GLP-1 secretion in animal models (43). Amgen demonstrated that multiple ligand binding pockets exist on FFA1, comprising of up to two allosteric sites as well as the FFA binding orthosteric site (40). One allosteric site is targeted by compounds such as AM-837 and TAK-875 while the second allosteric site is a target for receptor agonists such as AM-1638 that act as full agonists. Consequently, positive co-operativity was shown between either AMG-837 or AM-1638 in conjunction with natural FFA ligands in cell based assays measuring second-messenger generation, as well as primary cell based assays, with positive co-operativity also reported between AMG-837 and AM-1638 during an oral glucose tolerance test in a diabetic rodent model (40).

## FFA1 Agonists: Ongoing FFA1 Drug Programs and Future Challenges

Although no issues were raised regarding safety and tolerability during Phase I and II trials, Fasiglifam was recently withdrawn from phase III trials due to potential liver toxicity (43) (Table 1). Similarly, Amgen and Eli Lilly removed AMG-837 and LY2881835 (Table 1; Figure 2), respectively from Phase I clinical trials due to concerns over toxicity (43). However, the pre-clinical and clinical data generated using Fasiglifam provides a strong rationale and validation for further studies into the potential use of FFA1 agonism as a novel treatment for T2D. Currently, Japan Tobacco are conducting Phase II clinical trials with their FFA1 agonist candidate, JTT-851 and Piramal have begun Phase I clinical trials on their FFA1 agonist, P11187 (43) (Table 1). Daiichi Sanyko also recently described 3-aryl-3-ethoxypropanoic acids as orally active FFA1 agonists that improve insulin secretion and glucose homeostasis in rats (44). Additionally, FFA1 agonists developed by Astellas are reported to have beneficial effects on glucose homeostasis in diabetic mouse models (45, 46). Sanofi and Boehringer-Ingelheim are also reported to have FFA1 agonist programs under development (43). In an academic context, the University of Southern Denmark have developed 4-(benzylamine)hydrocinnamic acid FFA1 agonists such as TUG-469 (47, 48) and 4-alkyne hydrocinnamic acid FFA1 agonists, including TUG-424 and TUG-770 (49–51) (Table 1). Within these programs, several strategies have been followed to reduce compound lipophilicity (48, 52, 53). Consequently, TUG-770 (Figure 2) has recently been described as a highly potent FFA1 agonist with favorable physicochemical and pharmacokinetic properties, improving glucose tolerance in diet-induced obese mice. This effect did not desensitize, being fully maintained after 29 days of chronic dosing (49).

## FFA4

FFA4, a G_q_-coupled GPCR activated by LCFAs, including α-linolenic acid (α-LA) and docosahexaenoic acid (DHA) (Table 1), is expressed in enteroendocrine cells, lung, brain, white adipose tissues, heart, and liver (4). Within adipose tissue, FFA4 gene expression is upregulated following a HFD and FFA4 activation in adipocytes is associated with increased adipogenesis and glucose uptake (54–56), suggesting that FFA4 activation may promote adiposity and obesity (Figure 1). However, mutation of FFA4 (p.R270H variant) is associated with an increased risk of obesity in European populations (although this variant is almost absent in a Japanese population), and young FFA4 null mice fed a HFD gained significantly more fat mass than their wild-type littermates, suggesting that FFA4 protects against diet-induced obesity (57). FFA4 agonism is also commonly associated with improved insulin sensitivity, with FFA4 null mice reported to have increased fasting glucose and impaired responses to insulin and glucose tolerance testing (56, 57) (Figure 1). A number of these metabolic effects, such as increased insulin secretion, satiety, and improved glycemic control, have been attributed, at least in part, to FFA4-dependent incretin release from enteroendocrine cells, particularly GLP-1 (4, 54, 55, 58) (Figure 1). GLP-1 secretion was demonstrated both *in vitro* and *in vivo* using aLA as an agonist (58). Similarly, TUG-891 (Figure 2), a potent FFA4 agonist (see below), also increased GLP-1 secretion from STC-1 and GLUTag enteroendocrine cells (55). However, a recent study has questioned the significance of FFA4-mediated GLP-1 release (59). FFA4 also co-localizes with the orexigenic peptide, ghrelin, in duodenal cells *in vivo*, with recent studies showing that FFA4 activation inhibits ghrelin secretion (60, 61). An emerging role for FFA4 within pancreatic islets has also recently developed, with the pancreatic islets of diabetic and hyperglycemic individuals shown to have decreased levels of FFA4 mRNA and knockdown of FFA4 mRNA levels within islets demonstrated to attenuate the protective effects of the ω-3 fatty acid, eicosapentaenoic acid against palmitate-induced cell apoptosis (62) (Figure 1). FFA4 expression has also recently been detected in delta cells and alpha cells within the pancreas and was consequently linked with the inhibition of glucose-dependent somatostatin release and the regulation of glucagon secretion, respectively (63, 64) (Figure 1). Similar to FFA1, FFA4 is also expressed in taste buds and is linked with the regulation of taste preference although, again, the significance of this in relation to obesity and T2D remains to be clarified (65) (Figure 1).

## The Anti-Inflammatory Effects of FFA4

A recent study indicated that, in addition to the previously described insulin-sensitizing effects associated with GLP-1 release, improved systemic insulin sensitivity may also be associated with FFA4-mediated anti-inflammatory effects on macrophages (56) (Figure 1). In this study, FFA4 expression in macrophages was elevated in response to obesity and FFA4 activation decreased pro-inflammatory gene expression in M1 macrophages and increased expression of M2 anti-inflammatory genes with reduced macrophage infiltration of adipose tissues also observed in FFA4 null mice due to decreased chemotaxis (56). These anti-inflammatory effects are largely associated with FFA4-mediated recruitment of β-arrestin 2, a scaffold protein typically associated with receptor desensitization and internalization that is also implicated in the regulation of distinct signaling pathways (56, 66, 67). In the case of FFA4, β-arrestin 2 interacts with TAB1 that, in turn, inhibits lipopolysaccharide (LPS)- and tumor necrosis factor (TNF)-alpha-induced TAK1 stimulation, thereby blocking toll-like receptor 4 (TLR4) and the TNF-alpha inflammatory pathways (56, 66, 67). Interestingly, recent studies have also reported FFA4-mediated anti-inflammatory effects within the brain. In particular, FFA4 has been associated with the anti-inflammatory effects of ω-3 and ω-9 fatty acids in the hypothalamus, thereby reducing diet-induced inflammation and reducing body adiposity (68, 69) (Figure 1).

## Synthetic FFA4 Agonists

Initial synthetic FFA4 agonists, including GW9508, NCG21 (Figure 2), and NCG46 (Table 1), showed significant dual agonism at FFA1 (70). However, our groups have recently reported on TUG-891, a potent and selective FFA4 agonist (55, 71) (Table 1, Figure 2), although TUG-891 is significantly less selective for murine FFA4 compared to murine FFA1, potentially limiting its use in pre-clinical *in vivo* studies in mice (71). Recent modeling and mutational efforts have, however, clearly defined how TUG-891 interacts with FFA4 (72), information that will be invaluable in developing novel ligands with improved pharmacological properties for this receptor. To date, no FFA4 agonists have entered clinical trials although a number of FFA4 agonist programs are ongoing. For example, Banyu Pharmaceutical Co. Ltd, IRM LLC USA, Metabolex, Inc., Kindex Therapeutics, and Pharma Frontier Co., Ltd have all patented FFA4 agonists for the treatment of metabolic and inflammatory disease (66) (Table 1). Similarly, GSK has recently described a series of diarylsulfonamides as FFA4 agonists (73) and Metabolex has reported that their series of dihydrobenzofuran-based FFA4 agonists improved glucose homeostasis in mice, with moderate glucose-lowering effects in mice shown with a separate series of FFA4 agonists (66). Additionally, Kindex Therapeutics described beneficial effects in the treatment of obesity, inflammation, and metabolic disorders with alpha acids that were reported to act both as FFA4 agonists and also as partial PPARγ agonists (66).

## Metabolic Regulation by FFA2 and FFA3

High fiber intake protects against obesity and T2D via SCFA production, particularly butyrate, acetate, and propionate, from bacterial fermentation of dietary fiber in the large intestine (74). Moreover, modulation of gut microbiota using pre- and probiotics in both mice and humans regulates body weight, appetite, and glucose homeostasis (74). These SCFA-mediated beneficial effects on body weight and glucose homeostasis in HFD-fed mice are due, at least in part, to FFA2/FFA3-dependent mechanisms, including for example increased secretion of incretins, such as GLP-1, glucose-dependent insulinotropic polypeptide (GIP), and peptide YY (PYY) (74, 75). These receptors are activated by the SCFAs produced by fiber fermentation in the gut, with the human FFA2 ortholog preferentially activated by shorter SCFAs, such as acetate, whereas human FFA3 is activated preferentially by the longer SCFAs, with propionate being the most potent SCFA for both receptors, at least in human (4, 74–80) (Table 1; Figure 1). However, the relative potency and preference for various SCFAs appears to vary significantly across species (81, 82). SCFA-triggered secretion of GLP-1 was almost completely abolished in primary colonic cultures from FFA2 null mice and reduced, to a lesser extent, in mice lacking FFA3 (76). FFA2 is expressed in adipose tissue, intestine, islet cells, enteroendocrine cells, and immune cells while FFA3 is highly expressed in the small intestine, colon, and pancreas (4). FFA2 expression levels are also elevated in the skeletal muscle, liver, and adipose tissue of HFD-fed rodents, with FFA2 shown to regulate adipogenesis and adipocyte differentiation and inhibit lipolysis (83) (Figure 1).

Complete elucidation of the metabolic effects of FFA2 and FFA3 has, however, been complicated by conflicting results using FFA2 and FFA3 null mice. For example, in one study, HFD-fed FFA2 null mice display lower body fat mass and improved glucose control compared to wild-type mice, indicating a role for FFA2 antagonists in the treatment of T2D (84). Contrastingly, FFA2 null mice were also shown to be obese on a normal diet, with reduced insulin sensitivity and marked insulin resistance whereas adipocyte-specific overexpression of FFA2 resulted in lower body weight in a HFD study (85). Similarly, the loss of FFA3 either resulted in weight loss, obesity, or had no effect in different studies (86–88). Hence, the development of more potent and selective FFA2 and FFA3 agonists will hopefully facilitate the elucidation of the metabolic effects of FFA2 and FFA3 and ultimately provide future treatments for T2D. Several selective compound series are already known, especially for FFA2 (89) (Table 1). Small carboxylic acids derived from the natural SCFA ligands have shown appreciable and predictable selectivity but have low potency (80). Selective allosteric agonists of FFA2 were reported by Amgen to regulate lipolysis (e.g., AMG7703/4-CMTB, Table 1; Figure 2). However, the clinical use of these drugs was deemed to be limited due to low solubility and poor pharmacokinetics (90). Orthosteric FFA2 agonists and antagonists have also now been reported (82, 91) and used to demonstrate a role for this receptor in improved glucose uptake, decreased colon motility and contractility, increased GLP-1 secretion, and inhibiting leukocyte activation (81, 82, 89, 92). FFA3 agonists are even less developed although Arena Pharmaceuticals has reported a series of FFA3-selective compounds (89) (Table 1). Pharmacological characterization of compounds from this series demonstrated that individual members have diverse pharmacological properties, acting as intrinsic agonists and/or allosterically modulating the potency or efficacy of the response to SCFA propionate (93). Hence, although recent microbiota studies highlight quite elegantly the role that gut-derived SCFAs can play in the regulation of metabolism, there still remains a great demand for improved FFA2 and FFA3 agonists to fully unravel and define the consequences of activation of these receptors for metabolic health.

## Future Perspectives

The withdrawal of FFA1 agonists from clinical trials, particularly Fasiglifam, highlights the critical importance of establishing whether the adverse effects reported during these clinical trials are due to FFA1 agonism or the chemical structures of the particular FFA1 agonists. Additionally, as FFA2, FFA3, and FFA4 agonists further develop and hopefully enter clinical trials, it will be interesting to see if the same issues highlighted during FFA1 trials will also arise. Future research should also fully address the relative effects of partial and full FFA1 agonists, particularly in relation to allosterism in conjunction with natural FFA ligands. Additionally, dual agonists of FFA1 and FFA4 may have enhanced effects on insulin secretion and insulin sensitivity compared to selective FFA1 or FFA4 agonists alone. Similarly, co-therapeutic approaches involving FFA receptor agonists and current T2D therapies should be examined. For example, the FFA1 agonist, AS2575959 (Table 1), acts synergistically with a DPP-IV inhibitor to improve glucose homeostasis (45) and combination therapy with Fasiglifam and metformin displayed enhanced anti-diabetic effects in a diabetic rat model (94). Similarly, the FFA1 agonist, DS-1558 (Table 1), acts synergistically with exendin-4, a GLP-1 receptor agonist, to improve glucose homeostasis in diabetic mice (95). Clearly, there are a number of significant challenges ahead in the development of clinical treatments based on FFA receptor agonism. However, should these challenges be met, FFA receptor agonism may provide a novel and effective way to treat T2D.

## Conflict of Interest Statement

The authors declare that the research was conducted in the absence of any commercial or financial relationships that could be construed as a potential conflict of interest.

## Abbreviations

αLA, α-linolenic acid; DHA, docosahexaenoic acid; CNS, central nervous system; DPP-4, dipeptidyl peptidase-4; FFA, free fatty acid; FFA1–4, free fatty acid receptors 1–4; GIP, glucose-dependent insulinotropic polypeptide; GLP-1, glucagon-like peptide-1; GPCR, G protein-coupled receptor; GSIS, glucose-stimulated insulin secretion; HbA1c, hemoglobin A1c; HFD, high-fat diet; LCFA, long-chain fatty acid; LPS, lipopolysaccharide; MCFA, medium chain fatty acid; PYY, peptide YY; SCFA, short chain fatty acid; T2D, type 2 diabetes; TNF, tumor necrosis factor.
